# Jointly held care documents in child and adolescent mental health services: a systematic review

**DOI:** 10.3389/frcha.2026.1863944

**Published:** 2026-09-09

**Authors:** Neve Jones, James Roe, Josephine Holland

**Affiliations:** University of Nottingham, Nottingham, United Kingdom

**Keywords:** CAMHS, child and adolescent mental health, digital, electronic healthcare records, jointly held care documents

## Abstract

**Background:**

Young people accessing Child and Adolescent Mental Health Services (CAMHS) in the UK represent a digital native generation, but practices around digital care documents lag behind. Jointly held care documents could be a way of improving accessibility and inclusion of young people and parents/carers in care planning.

**Methods:**

Systematic searches of MEDLINE, Embase, CINAHL, PsycINFO, PsycArticles, King's Fund, Google Scholar, The Health Foundation, Social Care Online, Cochrane Library, Royal College of Psychiatrists, Web of Science and EconLit databases were conducted alongside grey literature searches. All eligible studies investigating the use of jointly held care documents in Child and Adolescent Mental Health Services were included. Risk of bias was assessed using an adapted version of the Hawker critical appraisal tool.

**Results:**

Seven studies, from research groups in the Netherlands, the USA and Norway were included. Existing studies have looked at perceptions of quality and engagement, facilitators and barriers, clinical effectiveness and adolescent and professional acceptability. Studies have used a range of methodologies and were generally of high quality, finding that jointly held care documents were mainly positively thought of by young people and parents/carers, but there was ambivalence from healthcare professionals around information sharing and the practicalities of co-creation.

**Conclusion:**

The current literature shows good acceptability of jointly held and co-created care documents amongst young people and parents. Barriers to implementation mainly exist amongst professionals.

**Systematic Review Registration:**

https://www.crd.york.ac.uk/PROSPERO/view/CRD420250652309.

## Background

Child and Adolescent Mental Health Services (CAMHS) are overwhelmed. Rising demand has exposed delays, fragmented care, and poor outcomes for people seeking support (1–3). Families often struggle to navigate unclear systems, with limited involvement in care planning despite its importance (4, 5). In response, national strategies including the NHS Long Term plan and Topol Review emphasize digital innovation to improve access, coordination and transparency (6, 7). Optimizing these systems is now critical.

Jointly held care documentation has been introduced internationally, with notable implementations in the Netherlands (EPR-Youth, Iuvenelis), Norway (adolescent-accessible EHR portals) and the USA (shared plans of care and patient portals) (8–14). Although these systems differ in structure, they share core elements: transparency of clinical information, opportunities for young people and parents/carers to contribute to care plans, and mechanisms for real-time communication across professionals. Comparable systems exist in adult mental health and physical healthcare, where collaboratively developed documentation had been associated with shared decision making, stronger therapeutic alliances and enhanced continuity of care.

In adult mental health and physical healthcare, shared decision making (SDM) is a collaborative process that strengthens therapeutic alliances, enhances service user autonomy, and improves engagement, even in complex mental health settings (15, 16). As a result of this, jointly held care documents such as co-produced care and crisis plans are produced (17). Evidence from older adult care planning (18) and recovery-oriented mental health coordination (19) demonstrates that collaboratively developed, transparent documentation strengthens decision-making, embeds service user goals, and supports therapeutic alliances across settings.

Within CAMHS, shared decision-making and collaborative documentation are notably underused, despite evidence that service user involvement improves satisfaction and trust (20). In adult mental health services, jointly held care documents have demonstrated value in enhancing transparency, continuity and embedding service user perspectives (21). However, developmental factors are central to how jointly held documentation is used in CAMHS. Decision-making capacity evolves across childhood and adolescence, autonomy increases with age, confidentiality becomes more salient, and preferences between young people and parents may diverge. Young people also often have wider care networks, including education and social care, meaning that jointly held documentation may support multi-agency coordination and reduce duplication across services (22).

Transitions between clinicians, teams and services are common in CAMHS, including discharge to primary care and transfer to adult mental health services. Shared documentation may help maintain continuity during these vulnerable points by preserving goals, preferences and care histories. These developmental, relational and organizational considerations highlight why youth-specific approaches are required rather than direct transfer of adult models. This is the first systematic review to examine existing models of jointly held care documentation and evaluate their potential to enhance shared decision-making and collaborative care within CAMHS.

## Methods

The systematic review was conducted in accordance with the Preferred Reporting Items for Systematic Reviews and Meta-Analyses (PRISMA) guidance (see supplementary material for the PRISMA checklist). The systematic review was registered with the International Prospective Register of Systematic Reviews (PROSPERO) in February 2025 (ID: CRD420250652309).

## Search strategy

Systematic searches of: MEDLINE, Embase, CINAHL, PsycINFO, PsycArticles, King's Fund, Google Scholar, The Health Foundation, Social Care Online, Cochrane Library, Royal College of Psychiatrists, Web of Science and Econlit databases were conducted alongside grey literature searches. The reference lists of relevant papers and policy articles were also searched during screening to identify any other potential studies. Initial searches covered all time points up to 14th February 2025

The searches combined search terms for jointly held care documents, digital, child and adolescent mental health and electronic healthcare records. The full search strategy is available in Supplementary Appendix 1. The search strategy included both open access and subscription-based databases. Studies were not restricted by access type. All included studies were published, peer-reviewed sources, and no unpublished data were used.

## Inclusion and exclusion criteria

Studies included were both randomized and non-randomized studies on the use of care documents or care records that patients/parents can access or edit. To be included, a study had to focus on mental health care for under-18-year-olds and required qualitative or quantitative primary data. Studies that were excluded were unrelated to care records or documentation, focused on another aspect of child and adolescent health or presented data only about over-18-year-olds. Investigations into care records or documentation that patients and/or their parents cannot view or modify were excluded.

## Screening

After the search, all titles and abstracts were uploaded to Rayyan Systematic Review software (23). Abstracts were independently considered for inclusion by two authors (NJ, JH or JR). If there was a dispute between two researchers on the inclusion of a study based on the abstract, a third researcher was consulted to establish a consensus.

All reference lists were checked by NJ, and where there were additional records identified, these were sent to a second author to decide if they should be included, if there was a conflict between the two decisions, the opinion of a third author was sought.

## Risk of bias

A modified version of the Hawker critical evaluation technique (24) was used to evaluate the studies because it prevented prejudice against studies presented in a non-traditional layout. Paul's modified version (25) was utilized. This tool gave researchers the freedom to evaluate studies using a variety of approaches, including qualitative ones. To guarantee score dependability, a second researcher used the critical appraisal tool independently, and then the scores were compared and agreed upon.

### Data extraction

Two researchers independently completed data extraction for each study. After completing this independently, the researchers collaborated to ensure all pertinent data had been retrieved.

### Data analysis

Due to the range of study designs and outcomes reported, meta-analysis was not felt to be appropriate for this data. A narrative synthesis method was therefore used to summarize the relevant studies.

## Results

## Study selection

**Figure 1:**
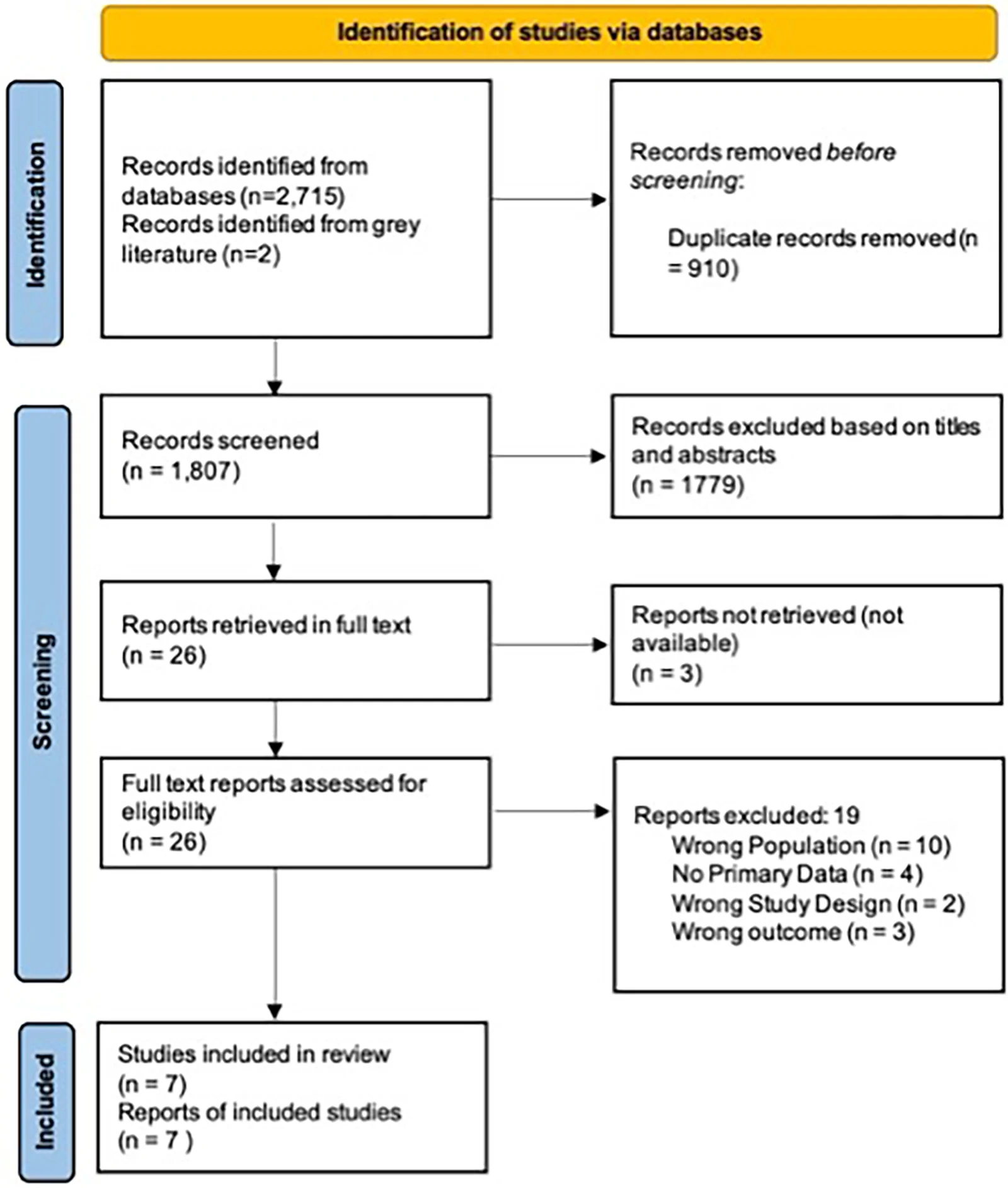
PRISMA flow chart (26).

The study selection process is shown in the PRISMA Flow Chart (Figure 1**)**.

A total of seven studies were included. Initial searches generated 2,715 results, 1,807 after duplicates were removed. Of these, 26 full texts were considered, from which 23 studies were included for review. Full text screening identified seven studies for inclusion. There were no studies which appeared to meet the inclusion criteria but were later excluded. The small number of eligible studies reflects the emerging nature of jointly held documentation within CAMHS.

Table 1 presents a summary of study characteristics and main findings.

**Table 1:** Study characteristics and main findings.

Study	Country	Aim	Methods	Participants	Intervention	Results	Quality score/ 36
Benjamins et al. (8), 2024	Netherlands	To assess whether and how using Patient-accessible electronic health records (PAEHR) contributes to perceived quality of care from a client's perspective	Qualitative Interviews	*N* = 20, Parents of children aged 0 to 16 years and adolescents aged ≥12 years, living in the North Veluwe region.	PAEHRs (Called Iuvenelis)	Parents and adolescents perceived that using Iuvenelis contributed to the quality of care because they felt better informed and more involved in the care process than before the introduction of Iuvenelis.	35
Benjamins et al. (9), 2023	Netherlands	To evaluate the implementation of EPR-Youth and to determine barriers and facilitators	Mixed Methods: integrated system data, questionnaires, process observations and focus group interviews	*N* = 5174, Target groups included adolescents, parents, professionals using EPR-Youth and key implementation stakeholders.	A fully accessible electronic patient record system for clients (EPR-Youth)	Client-portal acceptability was high across all clients, with adoption rates also high but varying by age group and educational level. Professionals expressed doubts about acceptability, appropriateness, and fidelity, largely due to limited system knowledge. Implementation barriers included the complexity of co-creation, unclear leadership, and legal concerns.	35
Guevara et al. (10), 2021	USA	To compare the effectiveness of care management with a patient portal against a patient portal alone for communication among children with ADHD.	Prospective randomized comparative effectiveness trial, Vanderbilt Parent Rating Scale (VPRS)	*N* = 273, 11 Primary care pediatric practices associated with a large children's hospital, from March 2016 to April 2019.	Shared decision-making (SDM) on electronic patient portals.	In multivariate models, VPRS scores decreased over time in both groups (Adjusted b = .015; 95% CI: 0.023 to 0.07), with no significant intervention-by-time effects between groups (Adjusted b = .000; 95% CI: 0.011 to 0.012). Children who received two or more care management sessions showed greater reductions in VPRS scores compared to those with fewer sessions.	32
Mann et al. (11), 2020	USA	To examine the impact of shared plans of care (SPoC), a care coordination tool, on healthcare utilization among children with special healthcare needs (CSHCN) and mental health conditions.	Poisson generalized linear mixed model.	Data on emergency department visits, hospitalizations, and primary care visits were collected through chart reviews of children with special healthcare needs (CSHCN).	Shared plans of care (SPoC)	Following SPoC implementation, there was a decrease in primary care visits, hospitalizations, and ED visits for CSHCN, with only the reduction in primary care visits reaching statistical significance. Notably, mental health care visits decreased by 39% after SPoC adoption.	25
Nielson et al. (12), 2024	Norway	To reach a consensus among authors of scientific papers on recommendations for health care professionals’ digital sharing of notes with adolescents in mental health care and to investigate whether staff at child and adolescent specialist mental health care clinics agreed with the recommendations.	A Delphi study and cross-sectional study: with authors of scientific papers to reach a consensus on recommendations	Delphi study: round 1, *n* = 27 Round 2 *n* = 21 17 recommendations Participants in the cross-sectional study were staff working at child and adolescent specialist mental health care clinics geographically spread in Norway *n* = 690	Electronic health record (EHR)	Of the 84 invited authors, 27 responded, reaching consensus on 17 recommendations related to digital sharing of notes with adolescents in mental health care. These recommendations addressed introducing digital access, writing notes, supporting healthcare professionals, and deciding when to withhold notes. Among 41 staff members at child and adolescent specialist mental health clinics, at least 60% agreed with all 17 recommendations. However, no consensus was reached on the appropriate age for adolescent access or the timing for sharing notes with parents.	32
Nielson et al. (13), 2023	Norway	To explore adolescents’ interest in and experiences with using patient portals in mental health care	Cross-sectional study	*N* = 53 Adolescents aged 12 to 18 receiving treatment at four specialist child and adolescent mental health services in Norway.	Electronic health record (EHR), Patient portals	Fifty-three adolescents (8.5%) aged 12–18 years (mean age 15) responded, with 64% expressing interest in using patient portals. Nearly half would share portal access with healthcare providers (48%) and designated family members (43%). One-third had previously used a patient portal: 28% to change an appointment, 24% to view medications, and 22% for communication.	29
Nielson et al. (14), 2023	Norway	To explore views on using patient portals for adolescents in mental health care among persons involved in and/or being affected by the intro- duction of a patient portal.	Qualitative Interviews	*N* *=* 14 healthcare providers in child and adolescent mental health care, young representatives from the user panel, or persons affiliated with an EHR-project introducing a patient portal.	Electronic health record (EHR), Patient portals	Informants’ opinions ranged from believing that patient portals could benefit adolescents in mental health care to concerns that they might negatively impact treatment. They emphasized the need for management to provide training and support for healthcare providers in using patient portals and telehealth.	26

### Perceptions of quality and engagement

Two studies investigated service users’ perceptions of clarity, utility and involvement in relation to shared care documentation. Benjamins et al. (8) examine parents’ and adolescents’ experiences with the luvenelis patient-accessible electronic health record (PAEHR), reporting that access enhanced their understanding of health information and promoted greater engagement in care processes. Similarly, Nielson et al. (13), in a survey of adolescents accessing mental health services, found that 64% expressed interest in using patient portals; nearly half indicated willingness to share access with healthcare providers, with many preferring selective disclosures.

The applied critical appraisal tool (25) found the quality of these studies to be overall good. Scores are shown in Table 1. While the first study employed in-depth qualitative interviews, it was limited by the under-representation of adolescents (*n* = 7). In contrast, the second study utilized a larger sample size, offering broader generalizability. Despite these methodological differences, both studies demonstrated a consistent direction of effect, indicating that access to health records and/or involvement in decision-making was associated with improved perceived care quality and engagement within children's mental health services.

#### Implementation barriers and facilitators

Both studies identified substantial barriers to implementing shared care documentation systems, notably the complexity of co-creation and the impact of organizational, professional and technical factors. Benjamins et al. (9) reported a high uptake of the ERP-Youth portal, with age-related variation in adoption, but noted challenges including limited professional system knowledge, privacy and legal concerns, complexity of co-creation, and insufficient leadership. Similarly, Neilson et al. (14) highlighted the need for structured training, ongoing managerial support, and attention to potential risks to therapeutic relationships.

The applied critical appraisal tool (25) found the quality of these studies to be overall good. Both studies exhibited methodological limitations, including sampling bias, insufficient longitudinal analysis and inadequate evaluation of implementation factors such as training and leadership dynamics.

#### Clinical effectiveness

Two studies examined the impact of digital interventions on service delivery and care coordination. Guevara et al. (10) reported that integration of patient portals with care management was associated with greater reductions in ADHD symptoms, particularly among families demonstrating higher levels of engagement. A second study (11) found that co-produced shared care plans were correlated with decreased utilization of primary care, emergency services, and mental health visits, suggesting improved care continuity and system efficiency.

The applied critical appraisal tool (25) found the quality of these studies to be overall good. The first study, despite its randomized design and validated measures, was constrained by engagement bias, reliance on patient-reported outcomes, and short follow-up. However, the second study, retrospective in nature, faces documentation bias and lacks control for confounding variables.

### Adolescent and professional acceptability

Feasibility and perceived value of adolescent access to digital health records were explored through both consensus-building and implementation research. Nielsen et al. (12) used a Delphi process to achieve agreement on 17 recommendations for adolescent access to clinical notes, although consensus was lacking regarding the appropriate access age and timing of parental viewing.

The applied critical appraisal tool (25) found the overall quality of these studies to be good. The first study used a Delphi process; it relied on expert consensus with limited youth representation and lacked agreement on key access parameters. The second study, in a secondary implementation phase, reported mixed professional perceptions without systematic quantification or longitudinal follow-up, limited generalizability and limited depth of analysis.

## Discussion

### Summary of findings

This systematic review identified seven studies examining jointly held care documentation within CAMHS. Two studies focused on perceived care quality, two on implementation barriers and facilitators, two on clinical effectiveness, and one on adolescent and professional acceptability. Several additional studies investigated shared documentation but were excluded due to targeting adult or non-CAMHS populations (27–29), highlighting both the relevance of this topic across healthcare and the growing interest in collaborative documentation in other specialties. Across the included studies, several cross-cutting themes emerged: adolescent autonomy, professional ambivalence, legal concerns and system-level barriers, which extend beyond primary outcome categories and warrant deeper exploration within youth mental health contexts.

### Interpretation of key findings

A central finding concerned perceptions of care quality and engagement. Access to jointly held documentation enhanced adolescents’ and parents’ understanding of care processes, promoted a sense of involvement, and supported more active participation in treatment. These experiential benefits are consistent with youth-centered care principles and align with broader evidence that engagement is a key determinant of CAMHS outcomes. Mechanisms underpinning these effects may include improved information sharing, clearer articulation of goals, strengthened therapeutic alliance, enhanced self-management and reduced fragmentation across clinicians and services.

### Alignment with existing literature

Overall, the findings broadly support existing literature on shared decision-making and collaborative documentation, consistent with adult-focused studies (16). Access to patient-held records was associated with improved engagement, understanding and perceived care quality among adolescents (8, 13). Similarly, reported clinical outcomes, such as reduced symptom severity and decreased service utilization (10, 11), mirror evidence from adult recovery-oriented care planning, where co-produced documentation enhances continuity and embeds service goals (19). Implementation barriers identified in this review, including limited system knowledge, co-creation complexity and weak leadership, also align with longstanding concerns about fragmented care pathways and organizational constraints within CAMHS (2). Together, these findings suggest that adult documentation models may offer a useful foundation for youth mental health services, provided they are adapted to developmental and contextual needs.

### Divergences from existing literature

Despite these areas of alignment, several divergences emerged. While existing research characterizes shared documentation as underutilized in CAMHS (20), this review highlights emerging interest and feasibility, particularly among those who expressed a clear desire for access and involvement (12, 14). This suggests that underutilization may stem more from systemic and professional barriers than from a lack of user engagement. Furthermore, the assumption that adult models can be readily transferred to youth settings is challenged by mixed professional views and unresolved ethical concerns, including selective disclosure and parental access (9). Although adult literature emphasizes the therapeutic value of transparency (21), CAMHS clinicians raised concerns that documentation may not always improve trust if not carefully implemented. These divergences underscore the need for youth-specific frameworks that account for developmental and relational complexities unique to CAMHS.

Developmental considerations help explain these mixed professional views. Adolescents often value autonomy and transparency, whereas younger children rely more heavily on parents or carers. Conflicts may arise when parental preferences differ from those of the young person, particularly around selective disclosure and confidentiality. These developmental dynamics highlight why adult documentation models cannot be directly transplanted into youth services without careful adaptation.

### Implications for practice

These findings have several implications for CAMHS practice. First, CAMHS rarely operates in isolation; young people often receive support across education, social care, primary care and voluntary sector services. Jointly held documentation may therefore facilitate multi-agency coordination by improving communication and reducing duplication. Second, continuity of care remains a major challenge, with young people frequently experiencing transitions between clinicians, teams and services, including discharge to primary care or transfer to adult mental health services. Shared documentation may help maintain continuity during these vulnerable transitions by preserving care plans, preferences and historical information. Third, potential risks must be considered, including increased clinician workload, misinterpretation of clinical notes, strain on therapeutic relationships, confidentiality complexities and unequal access to digital technology. These risks emphasize the need for careful implementation, training and safeguarding procedures.

### Strengths and limitations

A key strength of this study lies in its inclusivity and potential generalizability. The review encompassed papers examining a range of shared care systems, incorporating qualitative, quantitative and mixed methods designs. A comprehensive multi-database search strategy was employed, guided by predefined inclusion and exclusion criteria, and supplemented with grey literature to minimize publication bias and capture studies not disseminated through traditional academic channels. The inclusion of recent studies enhances the currency and relevance of the evidence on shared care documentation within CAMHS. However, several limitations must be acknowledged. The small number of eligible studies constrains the breadth of available evidence and limits the ability to draw firm conclusions. The heterogeneity of documentation models, outcome measures and study designs also makes direct comparison challenging and reduces the precision of synthesis. Variability in methodological quality and incomplete reporting across studies further limits confidence in the findings. Additionally, most included studies were conducted in digitally mature health systems, which may limit generalizability to jurisdictions governed by GDPR or those with different safeguarding, consent or parental-access requirements. Together, these limitations highlight the need for further empirical research before jointly held documentation can be widely implemented within CAMHS.

### Future directions

This systematic review has highlighted the paucity of research examining the impacts of shared care documentation within CAMHS. Further research exploring its longer-term effects on young people, families and services, including clinical and experiential outcomes, would help to build a more comprehensive understanding of its value and effectiveness. Research is also needed to identify youth-specific documentation frameworks that balance transparency, autonomy and safeguarding, particularly in contexts where parental access and selective disclosure present ethical challenges. Larger, methodologically robust trials should evaluate implementation processes, digital readiness, and multi-agency coordination, as well as potential risks such as clinician workload and confidentiality complexities. Comparative studies across different regulatory environments would further clarify how documentation systems can be adapted for safe and effective use in diverse CAMHS settings.

## Conclusion

This review demonstrates that jointly held care documentation can enhance engagement, transparency, and care continuity within CAMHS, echoing benefits observed in adult mental health services. While findings support the relevance of shared decision-making and collaborative documentation, they highlight unique challenges in your settings, including ethical concerns and professional hesitations. Tailored approaches and further research are needed to ensure effective, developmentally appropriate implementation. Tailored, developmentally appropriate approaches and further research are needed to ensure safe, effective implementation. Overall, jointly held documentation shows promise as a youth-centered tool for improving communication, involvement and continuity of care within CAMHS, but its successful adoption will depend on careful adaptation to the complexities of child and adolescent mental health practice.

## Data Availability

The datasets presented in this study can be found in online repositories. The names of the repository/repositories and accession number(s) can be found in the article/Supplementary Material.
