# Supplementary material for: Jointly held care documents in child and adolescent mental health services: a systematic review
Source: Front Child Adolesc Psychiatry. 2026 Sep 9;5:1863944. doi: 10.3389/frcha.2026.1863944 (PMC13597925; doi:10.3389/frcha.2026.1863944)
Supplement: Supplementary file 1 [file Datasheet1.docx]

**Library and Knowledge Services**

**Systematic Review Search Summary**

# ***Search strategies***

| **Platform and database**: Ovid MEDLINE® ALL <1946 to April 03, 2024> | | |
| --- | --- | --- |
| 1 | (child* or infant* or paediatric* or pediatric* or teen* or adolescen* or juvenile or "young person" or "young people" or minors or youth*).ti,ab. | 2524232 |
| 2 | exp Child/ or exp Pediatrics/ | 2223868 |
| 3 | exp Adolescent/ or exp Minors/ | 2240547 |
| 4 | 1 or 2 or 3 | 4421590 |
| 5 | exp Hospitals, Psychiatric/ or exp Psychiatric Department, Hospital/ or exp Mental Health Services/ | 134885 |
| 6 | (secure adj (unit* or hospital* or service* or facilit*)).ti,ab. | 594 |
| 7 | ((medium or low or regional) adj secur*).ti,ab. | 600 |
| 8 | ("psychiatric intensive care" or PICU or "locked rehabilitation").ti,ab. | 7321 |
| 9 | (mental or psychiatric or section*).ti,ab. | 1559326 |
| 10 | 5 or 6 or 7 or 8 or 9 | 1636522 |
| 11 | 4 and 10 | 404957 |
| 12 | ("out of area" or distance* or "out-of-area" or "distant from" or "far from" or "miles away" or "long way").ti,ab. | 827120 |
| 13 | (adult adj2 (setting* or ward* or unit*)).ti,ab. | 3275 |
| 14 | 12 or 13 | 830334 |
| 15 | 11 and 14 | 8987 |
| 16 | (placement* or place* or admit* or admission* or "sent to").ti,ab. | 1670515 |
| 17 | 15 and 16 | 995 |
| **Platform and database**: Ovid Embase <1974 to 2024 April 03> | | |
| 1 | (child* or infant* or paediatric* or pediatric* or teen* or adolescen* or juvenile or "young person" or "young people" or minors or youth*).ti,ab. | 3187082 |
| 2 | exp Child/ or exp Pediatrics/ or exp Juvenile/ or exp Infant/ | 4202588 |
| 3 | exp Adolescent/ or exp Adolescence/ | 1877603 |
| 4 | 1 or 2 or 3 | 5042682 |
| 5 | exp Psychiatric Department/ or exp Mental Hospital/ or exp Mental Patient/ | 63712 |
| 6 | (secure adj (unit* or hospital* or service* or facilit*)).ti,ab. | 985 |
| 7 | ((medium or low or regional) adj secur*).ti,ab. | 937 |
| 8 | ("psychiatric intensive care" or PICU or "locked rehabilitation").ti,ab. | 16107 |
| 9 | (mental or psychiatric or section*).ti,ab. | 2033870 |
| 10 | 5 or 6 or 7 or 8 or 9 | 2071236 |
| 11 | 4 and 10 | 488983 |
| 12 | ("out of area" or distance* or "out-of-area" or "distant from" or "far from" or "miles away" or "long way").ti,ab. | 1035634 |
| 13 | (adult adj2 (setting* or ward* or unit*)).ti,ab. | 4872 |
| 14 | 12 or 13 | 1040381 |
| 15 | 11 and 14 | 11973 |
| 16 | (placement* or place* or admit* or admission* or "sent to").ti,ab. | 2488158 |
| 17 | 15 and 16 | 1676 |
| **Platform and database**: ProQuest APA PsycInfo® <1806 to current> | | |
| S1 | ti,ab(child* OR infant* OR paediatric* OR pediatric* OR teen* OR adolescen* OR juvenile OR "young person" OR "young people" OR minors OR youth*) | 1046038 |
| S2 | MAINSUBJECT.EXACT.EXPLODE("Pediatrics") | 38272 |
| S3 | [S1] OR [S2] | 1051664 |
| S4 | MAINSUBJECT.EXACT.EXPLODE("Psychiatric Hospitalization") OR MAINSUBJECT.EXACT.EXPLODE("Psychiatric Hospitals") OR MAINSUBJECT.EXACT.EXPLODE("Psychiatric Clinics") OR MAINSUBJECT.EXACT.EXPLODE("Psychiatric Units") | 23137 |
| S5 | ti,ab(secure NEAR/1 (unit* OR hospital* OR service* OR facilit*)) | 1476 |
| S6 | ti,ab((medium OR low OR regional) NEAR/1 secur*) | 1601 |
| S7 | ti,ab("psychiatric intensive care" OR PICU OR "locked rehabilitation") | 659 |
| S8 | ti,ab(mental OR psychiatric OR section*) | 714068 |
| S9 | [S4] OR [S5] OR [S6] OR [S7] OR [S8] | 720156 |
| S10 | [S3] AND [S9] | 167893 |
| S11 | ti,ab("out of area" OR distance* OR "out-of-area" OR "distant from" OR "far from" OR "miles away" OR "long way") | 66474 |
| S12 | ti,ab(adult NEAR/2 (setting* OR ward* OR unit*)) | 4970 |
| S13 | [S11] OR [S12] | 71393 |
| S14 | [S10] AND [S13] | 1479 |
| S15 | ti,ab(placement* OR place* OR admit* OR admission* OR "sent to") | 323010 |
| S16 | [S14] AND [S15] | 185 |
| **Platform and database**: EBSCOhost CINAHL Plus with Full Text | | |
| S1 | TI ( child* OR infant* OR paediatric* OR pediatric* OR teen* OR adolescen* OR juvenile OR "young person" OR "young people" OR minors OR youth* ) OR AB ( child* OR infant* OR paediatric* OR pediatric* OR teen* OR adolescen* OR juvenile OR "young person" OR "young people" OR minors OR youth* ) | 940162 |
| S2 | (MH "Child+") OR (MH "Pediatrics+") OR (MH "Infant+") | 783403 |
| S3 | (MH "Adolescence+") OR (MH "Minors (Legal)") | 618195 |
| S4 | S1 OR S2 OR S3 | 1452355 |
| S5 | (MH "Hospitals, Psychiatric") OR (MH "Psychiatric Units") OR (MH "Mental Health Services+") | 102341 |
| S6 | TI ( secure N1 (unit* OR hospital* OR service* OR facilit*) ) OR AB ( secure N1 (unit* OR hospital* OR service* OR facilit*) ) | 892 |
| S7 | TI ( (medium OR low OR regional) N1 secur* ) | 254 |
| S8 | TI ( "psychiatric intensive care" OR PICU OR "locked rehabilitation" ) OR AB ( "psychiatric intensive care" OR PICU OR "locked rehabilitation" ) | 4272 |
| S9 | TI ( mental OR psychiatric OR section* ) OR AB ( mental OR psychiatric OR section* ) | 526237 |
| S10 | S5 OR S6 OR S7 OR S8 OR S9 | 590303 |
| S11 | S4 AND S10 | 170748 |
| S12 | TI ( "out of area" OR distance* OR "out-of-area" OR "distant from" OR "far from" OR "miles away" OR "long way" ) OR AB ( "out of area" OR distance* OR "out-of-area" OR "distant from" OR "far from" OR "miles away" OR "long way" ) | 106219 |
| S13 | TI ( adult N1 (setting* OR ward* OR unit*) ) OR AB ( adult N1 (setting* OR ward* OR unit*) ) | 2696 |
| S14 | S12 OR S13 | 108881 |
| S15 | S11 AND S14 | 3213 |
| S16 | TI ( placement* OR place* OR admit* OR admission* OR "sent to" ) OR AB ( placement* OR place* OR admit* OR admission* OR "sent to" ) | 423680 |
| S17 | S15 AND S16 | 362 |
| **Platform and database**: Clarivate Web of Science Core Collection | | |
| #1 | child* OR infant* OR paediatric* OR pediatric* OR teen* OR adolescen* OR juvenile OR "young person" OR "young people" OR minors OR youth* (Topic) | 3944745 |
| #2 | secure NEAR/1 (unit* OR hospital* OR service* OR facilit*) (Topic) | 4259 |
| #3 | (medium OR low OR regional) NEAR/2 secur* (Topic) | 10621 |
| #4 | "psychiatric intensive care" OR PICU OR "locked rehabilitation" (Topic) | 7976 |
| #5 | mental OR psychiatric OR section* (Topic) | 2501756 |
| #6 | #2 OR #3 OR #4 OR #5 | 2521634 |
| #7 | #1 AND #6 | 384714 |
| #8 | "out of area" OR distance* OR "out-of-area" OR "distant from" OR "far from" OR "miles away" OR "long way" (Topic) | 1290148 |
| #9 | adult NEAR/1 (setting* OR ward* OR unit*) (Topic) | 5424 |
| #10 | #8 OR #9 | 1295521 |
| #11 | #7 AND #10 | 4792 |
| #12 | placement* OR place* OR admit* OR admission* OR "sent to" (Topic) | 2652378 |
| #13 | #11 AND #12 | 552 |
| **Platform and database**: Cochrane Library | | |
| #1 | (child* OR infant* OR paediatric* OR pediatric* OR teen* OR adolescen* OR juvenile OR "young person" OR "young people" OR minors OR youth*):ti,ab (Word variations have been searched) | 258678 |
| #2 | MeSH descriptor: [Child] explode all trees | 81699 |
| #3 | MeSH descriptor: [Pediatrics] explode all trees | 1043 |
| #4 | MeSH descriptor: [Adolescent] explode all trees | 136681 |
| #5 | MeSH descriptor: [Minors] explode all trees | 15 |
| #6 | {OR #1-#5} | 360956 |
| #7 | (secure NEXT (unit OR hospital OR service OR facility)):ti,ab (Word variations have been searched) | 91 |
| #8 | (((medium OR low OR regional) NEAR/2 secur*) OR "psychiatric intensive care" OR PICU OR "locked rehabilitation"):ti,ab | 1186 |
| #9 | (mental OR psychiatric OR section):ti,ab (Word variations have been searched) | 119671 |
| #10 | MeSH descriptor: [Hospitals, Psychiatric] explode all trees | 304 |
| #11 | MeSH descriptor: [Psychiatric Department, Hospital] 2 tree(s) exploded | 130 |
| #12 | MeSH descriptor: [Mental Health Services] explode all trees | 9450 |
| #13 | {OR #7-#12} | 128075 |
| #14 | #6 AND #13 | 31242 |
| #15 | "out of area" OR distance* OR "out-of-area" OR "distant from" OR "far from" OR "miles away" OR "long way" OR (adult NEAR/2 (setting OR ward OR unit*)):ti,ab (Word variations have been searched) | 28862 |
| #16 | #14 AND #15 | 558 |
| #17 | (placement* OR place OR admit OR admission OR "sent to"):ti,ab (Word variations have been searched) | 134272 |
| #18 | #16 AND #17 | 128 |
| **Platform and database**: ProQuest EconLit (1886 to current) | | |
| S1 | ti,ab(child* OR infant* OR paediatric* OR pediatric* OR teen* OR adolescen* OR juvenile OR "young person" OR "young people" OR minors OR youth*) | 47601 |
| S2 | ti,ab(secure NEAR/1 (unit* OR hospital* OR service* OR facilit*)) | 35 |
| S3 | ti,ab((medium OR low OR regional) NEAR/2 secur*) | 671 |
| S4 | ti,ab("psychiatric intensive care" OR PICU OR "locked rehabilitation") | 1257 |
| S5 | ti,ab(mental OR psychiatric OR section*) | 36498 |
| S6 | [S2] OR [S3] OR [S4] OR [S5] | 38419 |
| S7 | [S1] AND [S6] | 2189 |
| S8 | ti,ab("out of area" OR distance* OR "out-of-area" OR "distant from" OR "far from" OR "miles away" OR "long way") | 21205 |
| S9 | ti,ab(adult NEAR/2 (setting* OR ward* OR unit*)) | 111 |
| S10 | [S8] OR [S9] | 21314 |
| S11 | [S7] AND [S10] | 52 |
| S12 | ti,ab(placement* OR place* OR admit* OR admission* OR "sent to") | 49024 |
| S13 | [S11] AND [S12] | 2 |
